# Habitual Chocolate Consumption May Increase Body Weight in a Dose-Response Manner

**DOI:** 10.1371/journal.pone.0070271

**Published:** 2013-08-07

**Authors:** James A. Greenberg, Brian Buijsse

**Affiliations:** 1 Department of Health and Nutrition Sciences, Brooklyn College of the City University of New York, Brooklyn, New York, United States of America; 2 Department of Epidemiology, German Institute of Human Nutrition Potsdam-Rehbruecke, Nuthetal, Germany; CUNY, United States of America

## Abstract

**Objective:**

Habitual chocolate intake was recently found to be associated with lower body weight in three cross-sectional epidemiological studies. Our objective was to assess whether these cross-sectional results hold up in a more rigorous prospective analysis.

**Methods:**

We used data from the Atherosclerosis Risk in Communities cohort. Usual dietary intake was assessed by questionnaire at baseline (1987–98), and after six years. Participants reported usual chocolate intake as the frequency of eating a 1-oz (∼28 g) serving. Body weight and height were measured at the two visits. Missing data were replaced by multiple imputation. Linear mixed-effects models were used to evaluate cross-sectional and prospective associations between chocolate intake and adiposity.

**Results:**

Data were from 15,732 and 12,830 participants at the first and second visit, respectively. More frequent chocolate consumption was associated with a significantly greater prospective weight gain over time, in a dose-response manner. For instance, compared to participants who ate a chocolate serving less often than monthly, those who ate it 1–4 times a month and at least weekly experienced an increase in Body Mass Index (kg/m2) of 0.26 (95% CI 0.08, 0.44) and 0.39 (0.23, 0.55), respectively, during the six-year study period. In cross-sectional analyses the frequency of chocolate consumption was inversely associated with body weight. This inverse association was attenuated after excluding participants with preexisting obesity-related illness. Compared to participants without such illness, those with it had higher BMI and reported less frequent chocolate intake, lower caloric intake, and diets richer in fruits and vegetables. They tended to make these dietary changes after becoming ill.

**Conclusions:**

Our prospective analysis found that a chocolate habit was associated with long-term weight gain, in a dose-response manner. Our cross-sectional finding that chocolate intake was associated with lower body weight did not apply to participants without preexisting serious illness.

## Introduction

Obesity is a serious public health problem [1] that has stimulated interest in three recent cross-sectional epidemiological studies which found chocolate intake to be associated with lower body weight. The first analyzed a sample of 15,023 U.S. adults and found a trend toward chocolate intake being significantly associated with lower Body Mass Index (BMI in Kg/m^2^) [2]. The second found more frequent chocolate intake to be significantly linked to lower BMI in 1,018 Californian adults free of known cardiovascular disease (CVD) and diabetes [3]. The third studied 1,259 elderly Finnish men and found that those who preferred chocolate to non-chocolate candy had lower BMI [4]. Logically, the high caloric density of chocolate should increase the risk of weight gain, so more rigorous prospective epidemiological studies are needed to confirm these cross-sectional results.

Our objective was to assess the prospective and cross-sectional associations between chocolate intake and body weight. We hypothesized that there would be a positive association between chocolate intake and that differences between the prospective and cross-sectional results would be due to obesity-related illness. Such illness could tend to motivate subjects to decrease their body weight by decreasing their consumption of energy dense foods such as chocolate. As described herein, we used data from the Atherosclerosis Risk in Communities Study [5] cohort (http://www2.cscc.unc.edu/aric/) for this analysis.

## Materials and Methods

### Study Population

ARIC is a prospective cohort of participants (55.2% women) aged 45–64 years derived from 16,000 randomly selected persons in four United States communities in North Carolina, Minnesota, Maryland and Mississippi [6]. ARIC participants were examined at visit 1 in 1987–89, and then again in 1990–92 (visit 2), 1993–95 (visit 3), and 1996–98 (visit 4). This study used the limited access data set with obtained from the Collaborative Studies Coordinating Center in the Department of Biostatistics at the University Of North Carolina at Chapel Hill.

### Assessment of Dietary and Chocolate Intake

Usual dietary intake over the past year was assessed at visit 1 and ∼6 years later at visit 3 using a 66-item semi-quantitative, interviewer-administered food-frequency questionnaire (FFQ). This questionnaire was a modified version of the 61-item food-frequency questionnaire of Willett and colleagues [7]. Participants reported the frequency of consumption of specific foods and beverages in nine predefined categories, ranging from never or <1 per month to ≥6 times per day. Standard portion sizes were given as a reference for intake estimation. Chocolate consumption was assessed by one item that asked about the frequency of eating a 1-oz (∼28 g) portion of a chocolate bar or pieces. This item did not differentiate between different types of chocolate (white, milk, plain).

For our analyses we condensed the original nine categories of chocolate intake into three, based on the frequency of consumption of a 1-oz serving: <1/month, 1–4/month, and ≥1/week. We also converted the nine categories into a continuous variable by translating the consumption frequency at the midpoint of each interval into the number of oz of chocolate consumed per day. For instance the 5th category, 5–6 1-oz servings/week, was converted to 0.79 oz of chocolate per day.

### Outcome Variable

At all four visits, anthropometrics were assessed with the participant wearing a scrub suit and no shoes and with an empty bladder. Body weight was measured to the nearest pound (lb) using a beam balance. Body weight in pounds was then converted to kilograms (kg  = 0.4536 lb). Height was measured to the nearest centimeter with the participants looking straight ahead, standing erect on the floor, with heels together and back pressed against the wall where a vertical metal centimeter rule was mounted. BMI was calculated by dividing body weight in kilograms by the square of height in meters (kg/m^2^). BMI was the outcome variable in all our analyses.

### Confounders

Both the prospective and cross-sectional models were adjusted for potential confounders using three different regression models. Our objective was to provide information on the effects of three different types of confounders: demographic and ethnic variables (model A); socio-economic and behavioral variables (model B); and dietary intake (model C). Model A included age (years), race (black, non-black), and sex (male, female). Model B further adjusted for education (grade school or less; some high school; high school graduate; vocational school; some college; graduate or professional school), alcohol intake (0, >0 to <75, ≥75 to <150, and ≥150 g/week), smoking status (never, former, current <20 cigarettes/day, and current ≥20 cigarettes/day), prevalent obesity-related illness (yes/no, based on self-reported physician diagnosed myocardial infarction, stroke, diabetes, or cancer), energy intake (kcal/day) and physical activity (a continuous variable that combined activity indices for work, sports and leisure derived from a modified version of the Baecke questionnaire [8]. Model C further included the dietary level of vegetables, fruit, and total fat, based on the residual method [9]. The quadratic version of age was included whenever it significantly improved model fit.

The public-access ARIC dataset that was used for the current analysis did not contain a variable indicating the center in which subjects were interviewed and observed, so we were unable to control for center effects in our models.

### Statistical Methods

In prospective analyses we used a linear mixed-effects model with BMI as the dependent variable to assess whether chocolate consumption was related to changes in BMI over time. This model is suitable for analyzing repeated-measure data in which sequential measures are correlated [10]. Chocolate intake was modeled as a continuous variable, and as a categorical variable with the lowest intake frequency (<1 per month) as referent. We modeled visit as a continuous variable so as to estimate the mean change in BMI per 6 years (the period between visit 1 and 3). Chocolate intake, time (study visit), and an interaction term between chocolate and time were independent variables. To account for the correlations between repeated measures on the same participant, the intercept and coefficient of time were allowed to randomly vary between participants. The random intercept accounts for variations in BMI across participants that are independent of the secular changes in their BMI changes across visits. We used a compound symmetry covariance structure as it yielded good model fit with fewest parameters [10]. We adjusted for body weight and waist-to-hip ratio at visit 1 to account for differences in baseline weight and adiposity. We used data at visit 1 and updated the outcome, exposure and all confounder variables at visit 3. We were not able to use any data at visits 2 and 4 because food -frequency data, including the chocolate exposure variable, were not collected at these visits.

For the cross-sectional analysis, data from both visit 1 and 3 were combined in a random intercept model. This model allows for combination of data from participants at sequential visits, and analysis of correlated data [11]. Chocolate intake was modeled as a continuous variable - as had been done in all prior publications [2]–[4].

We performed multiple imputation to replace missing values of the outcome, exposure and confounder variables using the Markov chain Monte Carlo method [12] and generated eight imputed datasets. The models used to impute variables at visit 1 and 3 only contained data from visit 1 and 3, respectively, in order to preserve interaction effects due to time. No variable used in our analyses had more than 3% of values missing at visit 1 or 3. For our outcome variable, BMI, it was 0.15%; for our exposure variable, frequency of chocolate intake, it was 0.44%. For dietary variables such as total fat intake the missing rate was the highest, 2.82%. The Log-likelihood test was used to assess model fit and significance tests were two-sided. IBM SPSS Statistics (v. 20, IBM Corp., Armonk, NY) was used for the analyses for Table 1, and all other analyses were conducted with SAS (v. 9.3, SAS Institute Inc., Cary, North Carolina). This manuscript follows the Strengthening the Reporting of Observational Studies in Epidemiology (STROBE) recommendations [13], [14].

**Table 1:** Characteristics a of Participants in Different Categories of Chocolate Consumption at Visit 1 in the ARIC b Cohort.

	Frequency of Consumption of a 1 oz Serving of Chocolate				
	<1/month	1–4/month	2–6/week	≥1/day	
Characteristics	(N = 5,084)	(N = 6,256)	(N = 2,805)	(N = 1,030)	*P-value* c
Age (yrs)	54.7 (5.8)	54.0 (5.7)	53.9 (5.7)	53.7 (5.6)	<.0005
Chocolate (servings/day)	0.00 (0.00)	0.10 (0.04)	0.50 (0.14)	1.26 (0.73)	NA
BMI (Kg/m^2^)^d^	27.8 (5.6)	27.7 (5.3)	27.5 (5.2)	27.3 (5.2)	.11
race (% Black)	39.0	22.7	15.2	18.3	<.0005
sex (% male)	44.6	43.7	47.8	44.4	.004
Ever smoker (%)	57.0	57.4	60.2	64.1	<.0005
Alcohol Intake (gms/week)	47.8 (109.0)	41.3 (93.0)	39.6 (83.8)	33.4 (81.8)	<.0005
Educational Level^e^	3.34 (1.60)	3.66 (1.47)	3.69 (1.40)	3.48 (1.45)	<.0005
Basic	29.6	20.6	18.6	25.0	<.0005
Intermediate	36.8	41.8	44.6	44.3	
Advanced	33.6	37.6	36.8	30.8	
Physical Activity^e^	6.86 (1.51)	7.01 (1.42)	7.09 (1.40)	6.97 (1.51)	<.0005
Dietary Calories (Kcal/day)	1,455 (547)	1576 (559)	1,858 (625)	2126 (680)	<.0005
Daily Fat (gm)f	48.2 (12.5)	50.9 (12.1)	54.0 (12.9)	56.7 (14.4)	<.0005
Vegetables (servings/day)	1.58 (1.09)	1.36 (0.94)	1.27 (0.96)	1.13 (0.96)	<.0005
Fruit (servings/day)^f^	1.54 (1.38)	1.38 (1.20)	1.27 (1.14)	1.23 (1.42)	<.0005

a Data are given as mean (SD) for continuous variables and as percentages for categorical variables. Data are for participants with no missing values for any of the characteristics in this table. N is the number of such participants.

b ARIC, Atherosclerosis Risk in Communities Cohort.

c Based on the analysis of variance, Kruskal-Wallis test, or Chi-square test.

d BMI, Body Mass Index, calculated as measured weight in kilograms divided by the square of measured height in meters.

e Educational level and Physical activity were quantified by ARIC researchers. Physical activity was based on exercise, work and leisure activities.

f Adjusted for daily caloric intake using the residual method [9].

### Ethics Statement

The study protocol was approved by the Brooklyn College Institutional Review Board.

## Results

### Primary Analyses

Compared to participants who ate chocolate less frequently at visit 1, those who consumed it more frequently were more likely to be younger, thinner, white, female, and smokers, to consume less alcohol, and to have diets rich in calories and fat, and low in vegetables and fruit (Table 1). Within the 6-year period between visit 1 and 3, BMI increased an average of 0.44 kg/m^2^ (95% CI: 0.80, 0.87).

In our main cross-sectional and prospective analyses (results in Table 2 and 3, respectively), we used multiple-imputation to replace missing values of the outcome, exposure and confounder variables for all participants who attended both visit 1 and visit 3. There were 16,000 persons in the original sample selected by ARIC researchers [6]. Of these 16,000 participants, 15,732 contributed data at visit 1 and were in the limited-access dataset used in this study. In our main analyses, we included visit-1 data from these 15,732 participants. Of the 15,732 participants, 735 died prior to visit 3 and 2,167 did not attend visit 3. This left 12,830 participants who contributed visit-3 data to our main analyses. There were therefore a total of 28,562 participants who contributed data to our main analyses, and the follow-up rate of the 15,732 visit-1 participants at visit 3 was 81.6%.

**Table 2:** Chocolate Intake and Prospective Change in Body Mass Index (BMI) During a Six-year Period Between Visits 1 & 3 in the ARIC^a^ Cohort.

	FREQUENCY OF EATING A 1 oz SERVING OF CHOCOLATE^b^									A 1 oz (∼28 g) EXTRA			P for linear trend^h^	P forquad-ratictrend^h^
	<1/MONTH			1–4/MONTH			≥1/WEEK			DAILY SERVING				
	N^c^ at	N at		N at	N at		N at	N at		N at	N at			
Model	Visit 1	Visit 3	BMI Change^d^	Visit 1	Visit 3	BMI Change	Visit 1	Visit 3	BMI Change	Visit 1	Visit 3	BMI Change		
A^e^	5,29l4	4075	0 (referent)	3,718	3,106	0.22 (0.04, 0.40)	6,720	5,649	0.33 (017, 0.48)	15,732	12,830	0.13 (−0.01, 0.06)	.060	.267
B^f^	5,294	4075	0 (referent)	3,718	3,106	0.25 (0.07, 0.42)	6,720	5,649	0.36 (0.21, 0.52)	15,732	12,830	0.14 (0.00, 0.07)	.048	.257
C^g^	5,294	4075	0 (referent)	3,718	3,106	0.26 (0.08, 0.44)	6,720	5,649	0.39 (0.23, 0.55)	15,732	12,830	0.19 (0.04, 0.15)	.014	.499

a ARIC, Atherosclerosis Risk in Communities Cohort.

b Frequency of chocolate intake was assessed by means of a semi-quantitative food frequency question.

c N is the number of participants who provided data values in each of the categories of chocolate intake at visit 1 and 3. Missing values of outcome, exposure and confounder variables were replaced by multiple imputation.

d BMI change is the change in BMI (kg/m^2^) during the three year period between sequential visits among participants in a particular chocolate intake category, compared to participants who ate chocolate monthly or less frequently (referent category). Data are presented as mean (95% confidence interval) estimated by means of a linear mixed effects model, in which the exposure variable and confounders were updated at visit 2, 3 & 4 using the value assessed at the visit or the latest available value. Food-frequency variables, including the exposure variable were corrected for intra-individual variation by using the mean of the two values assessed at visit 1 and 3.

e Model A - adjusted for age, age squared, race (non-black, black), sex (male, female) & baseline covariates - body weight and waist-to-hip ratio;

f Model B - adjusted for model A variables plus alcohol intake (0, 0–75, 75–150 & > = 150 g/week), smoking (never, former, current <20/day, current ≥20/day), education (grade school or less; some high school; high school graduate; vocational school; some college; graduate or professional school), prevalent illness (preexisting, physician diagnosed heart attack, stroke, diabetes, or cancer), and dietary caloric intake.

g Model C - adjusted for model B variables plus energy-adjusted dietary vegetable, fruit and fat levels.

h Tests for linear and quadratic trend were performed by putting the linear and quadratic versions, respectively, of chocolate intake in the model.

**Table 3:** Cross-sectional Association between Chocolate Intake^a^ and Body Mass Index (BMI)^b^ in the ARIC^c^ Cohort.

	N at	N at	Change in BMI for an extra	P for linear trend^l^	P for quadratic trend^l^
MODEL	Visit 1	Visit 3	1 oz (∼28 g) daily serving		
***ALL PARTICIPANTS***					
A^e^	15,732	12830	−0.01 (−0.15, 0.13) k	.863	.391
B^f^	15,732	12830	−0.03 (−0.18, 0.12)	.689	.191
C^g^	15,732	12830	−0.16 (−0.31, −0.01)	.043	.413
***PARTICIPANTS WITH PREVALENT OBESITY-RELATED ILLNESS*** h					
A^e^	2,835	2,705	−0.65 (−1.02, −0.28)	<.001	.568
B^f^	2,835	2,705	−0.63 (−1.01, −0.26)	<.001	.373
C^g^,j	2,835	2,705	−0.71 (−1.08, −0.33)	<.001	.380
***PARTICIPANTS WITHOUT PREVALENT OBESITY-RELATED ILLNESS***					
A^e^	13,027	9,995	0.18 (0.03, 0.33)	.02	.123
B^f^	13,027	9,995	0.10 (−0.06, 0.26)	.226	.241
C^g^,j	13,027	9,995	−0.04 (−0.20, 0.13)	.665	.529

a Frequency of chocolate intake was assessed by means of a semi-quantitative food frequency question.

b BMI, Body Mass Index - weight in Kg divided by height in m squared.

c ARIC, Atherosclerosis Risk in Communities Cohort.

d N is the number of participants who provided data values at visit 1 and 3. Missing values of the outcome, exposure and confounder variables were replaced by multiple imputation.

e Model A - adjusted for age, age squared, race (non-black, black), sex (male, female);

f Model B - adjusted for model A variables plus alcohol intake (g/week), smoking status (never, former, current <20/day, current ≥20/day), educational level (grade school or less; some high school; high school graduate; vocational school; some college; graduate or professional school), prevalent illness (preexisting, physician diagnosed heart attack, stroke, diabetes, or cancer), and dietary caloric intake.

g Model C - adjusted for model B variables plus energy-adjusted dietary vegetable, fruit and fat levels.

h Prevalent obesity-related illness was based on yes/no responses to questions about the existence of physician diagnosed heart attack, stroke, diabetes, and cancer.

j the smoking confounder was a continuous variable in these two models.

k Data are presented as mean and 95% confidence interval, based on a random intercept model using combined data from ARIC visit 1 and 3 (see Statistical Methods).

l P for linear trend was assessed with the linear version of the exposure variable in the model. P for quadratic trend was assessed with both linear and quadratic versions of the exposure variable in the model.

Differences between respondents and non-respondents in confounder values at baseline were almost all less than 10%, and most were less than 5%. There were several exceptions. For instance, the proportion of current smokers with a > = 20 cigarette/day habit was 11.8% higher, and the proportion of participants with grade school or less education was 10.7% higher among non-respondents. The results of a sensitivity analysis of the effects of imputing missing values for non-respondents are reported in Secondary Analyses.

Table 2 shows the prospective mean changes in BMI from visit 1 to visit 3 (a 6-year period) according to categories of chocolate intake, with the lowest category (<1/month) being the referent. As described in Statistical Methods, these results used data with imputed values for missing data for participants who attended visit 1 and 3. The number of participants who provided data values in the <1/mo chocolate intake category was 5,294 and 4075 in visit 1 and 3, respectively. In the 1–4/mo category there were 3,718 and 3,106, such participants, in the >1/week category there were 6,720 and 5,649 such participants, and in total there were 15,732 and 12,830 such participants, respectively. More frequent chocolate intake was associated with a greater increase in BMI over time, and this trend was significant for the two higher levels of confounder adjustment. The BMI increases were higher in participants with higher frequencies of chocolate consumption a dose-response manner.

The cross-sectional analysis in the total sample revealed that chocolate consumption was associated with significantly lower BMI only after adjustment for intakes of vegetables, fruit, and total fat (Table 3). For this analysis 15,732 and 12,830 participants provided data at visit 1 and 3, respectively.

### Secondary Analyses

To investigate whether the inverse cross-sectional association may be explained by dietary changes due to obesity-related illness, we modeled an interaction term between chocolate intake and prevalent illness. This term was significant (p = .005) and we found that the significant inverse association between chocolate intake and BMI was restricted to participants with obesity-related illness and not present in participants without such illness (Table 3). There were 2,835 and 2,705 participants with prevalent serious illness, who provided data values at visit 1 and 3, respectively. There were 13,027 and 9.995 participants without prevalent serious illness who provided data values at visit 1 and 3, respectively.

We also made a comparison of the change in characteristics between visit 1 to 3 in participants who reported a first obesity-related illness between visit 1 and 3 with those who did not report such illness (Table 4 and 5). Compared to obese participants without self reported incident illness between visit 1 and 3, obese participants with new obesity-related illness reported significant greater decreases in chocolate (∼37%), dietary fat (∼4.5%) intake and BMI (∼2.6%), and greater increases in fruit (∼20%) and vegetable (∼17%) intake. Similar patterns were seen in the non-obese participants, though they were generally smaller in magnitude and only significant for fruit intake and BMI. We also compared the characteristics at visit 1 of participants with and those without self-reported prevalent obesity-related illness. Participants with the illness reported eating chocolate less frequently and consuming diets with more energy and richer in fruit and vegetables. They were also heavier than those without illness. For instance, among those with and without the illness, respectively, the mean intake of chocolate (servings/day) was 0.23 (95%CI: 0.22, 0.24) and 0.17 (0.16, 0.18), the mean intake of fruit (servings/day) was 1.37 (1.35, 1.39) and 1.58 (1.53, 1.63), and mean BMI (kg/m^2^) was 27.4 (27.4, 27.6) and 28.8 (28.6, 29.0).

**Table 4:** Changes in Characteristics of Obese Participants (BMI≥30 kg/m^2^)and Incident Obesity-related Illness^1^ between Visit 1 and 3 in the ARIC^2^ Cohort.

	INCIDENT OBESITY-RELATED ILLNESS BETWEEN VISIT 1 & 3							
	No (N = 1,992)			Yes (N = 453)			Yes-No	
Characteristic^3^	Visit 1	Visit 3	Visit 1 to 3	Visit 1	Visit 3	Visit 1 to 3	Difference	
Chocolate Intake	0.232 (.009)	0.235 (.011)	+0.004 (0.005)	0.227 (.018)	0.148 (.014)	−0.079 (.019)	−0.082 (.025)***	
Energy Intake	1636.8 (13.4)	1612.7 (13.5)	−24.1 (13.3)	1705.0 (30.4)	1632.2 (28.1)	−72.8 (27.8)	−48.7 (10.9)	
Fat Intake	60.86 (0.59)	57.19 (0.58)	−3.67 (0.58)	64.39 (1.34)	57.82 (1.23)	−6.58 (1.27)	−2.91 (1.35)*	
Vegetable Intake	1.49 (0.02)	1.62 (0.03)	+0.13 (0.03)	1.52 (0.05)	1.90 (0.07)	+0.38 (0.07)	+0.25 (0.06)***	
Fruit Intake	1.55 (0.03)	1.76 (0.03)	+0.21 (0.04)	1.49 (0.06)	2.00 (0.07)	+0.51 (0.08)	+0.30 (0.08)***	
Body Mass Index	34.18 (0.09)	35.31 (0.11)	+1.12 (0.06)	34.60 (0.19)	34.84 (0.23)	+0.24 (0.14)	−0.88 (0.14)****	

P-values (*<.05, **<.01, ***<.001, ****<.0001) are based on the two-sample t test of the difference between participants with and without incident obesity-related Illness in the difference in the characteristic between visit 1 and 3. Data are mean (standard deviation). Data are for participants with no missing values for any of the characteristics at visit1 or 3.

1 Obesity-related Illness was defined as self-reported physician diagnosed heart attack, stroke, diabetes, or cancer.

2 ARIC, Atherosclerosis Risk in Communities Prospective Cohort.

3 Units of measurement: chocolate intake - 1 oz servings/day; Daily Energy & Fat Intake - calories/day; Vegetable & Fruit Intake - 1/4 cup servings/day; Alcohol Intake - gm/week; Physical Activity - quantified by ARIC researchers based on the intensity, duration and frequency of activity during work, sports and leisure activities (see Materials and Methods); Body Mass Index - kg/m^2^.

P-values (*<.05, **<.01, ***<.001, ****<.0001) are based on the two-sample t test of the difference between participants with and without incident obesity-related illness in the difference in the characteristic between visit 1 and 3.

**Table 5:** Changes in Characteristics of Non-Obese Participants (BMI<30 kg/m^2^)and Incident Obesity-related Illness^1^ between Visit 1 and 3 in the ARIC^2^ Cohort.

	INCIDENT OBESITY-RELATED ILLNESS BETWEEN VISIT 1 & 3						
	No (N = 6,573)			Yes (N = 1,291)			Yes-No
Characteristic^3^	Visit 1	Visit 3	Visit 1 to 3	Visit 1	Visit 3	Visit 1 to 3	Difference
Chocolate Intake	0.232 (.004)	0.241 (.005)	+0.008 (0.006)	0.237 (.012)	0.226 (.016)	−0.011 (.016)	−0.019 (0.017)
Energy Intake	1618.4 (7.3)	1579.6 (7.3)	−38.7 (6.7)	1670.5 (21.0)	1599.7 (19.8)	−70.8 (20.9)	−32.0 (20.3)
Fat Intake	59.39 (0.33)	54.72 (0.32)	−4.68 (0.30)	61.66 (0.93)	55.24 (0.88)	−6.41 (0.90)	. −1.74 (0.90)
Vegetable Intake	1.45 (0.01)	1.59 (0.02)	+0.14 (0.02)	1.47 (0.04)	1.68 (0.05)	+0.21 (0.05)	+0.07 (0.05)
Fruit Intake	1.44 (0.02)	1.69 (0.02)	+0.25 (0.02)	1.43 (0.04)	1.81 (0.06)	+0.37 (0.05)	+0.13 (0.05)*
Body Mass Index	25.02 (0.03)	26.06 (0.04)	+1.04 (0.02)	25.58 (0.10)	26.31 (0.11)	+0.73 (0.07)	−0.31 (0.07)****

P-values (*<.05, **<.01, ***<.001, ****<.0001) are based on the two-sample t test of the difference between participants with and without incident obesity-related Illness in the difference in the characteristic between visit 1 and 3. Data are mean (standard deviation). Data are for participants with no missing values for any of the characteristics at visit1 or 3.

1 Obesity-related Illness was defined as self-reported physician diagnosed heart attack, stroke, diabetes, or cancer.

2 ARIC, Atherosclerosis Risk in Communities Prospective Cohort.

3 Units of measurement: chocolate intake - 1 oz servings/day; Daily Energy & Fat Intake - calories/day; Vegetable & Fruit Intake - 1/4 cup servings/day; Alcohol Intake - gm/week; Physical Activity - quantified by ARIC researchers based on the intensity, duration and frequency of activity during work, sports and leisure activities (see Materials and Methods); Body Mass Index - kg/m^2^.

P-values (*<.05, **<.01, ***<.001, ****<.0001) are based on the two-sample t test of the difference between participants with and without incident obesity-related illness in the difference in the characteristic between visit 1 and 3.

As a sensitivity analysis of the effects of our multiple-imputation of missing values, we repeated our analyses after not imputing any missing values, and instead performed a complete-cases analysis for participants who were at visit 1 and 3. For the complete-case analyses we excluded participants with unusually small or large values for dietary energy intake, defined as <600 or >4200 kcal/day for men and <500 or >3600 kcal/day for women [15]. There were 326 and 393 such participants at visit 1 and visit 3, respectively, leaving 27,843 participants without unusual values. We also excluded another 1,170 participants with missing values for our outcome, exposure or any confounder variable, leaving 26,673 participants who contributed data for these analyses - 8,684, 6,383 and 11,606 participants in the three chocolate-intake (1 oz servings) levels of <1/mo, 1–4/mo and ≥1/week, respectively. In “Statistical Methods” we have provided data on missing values for our outcome, exposure and confounder variables. The results were all very similar to those based on the multiply-imputed data. For instance, for the full-model prospective analysis (equivalent to 3rd row in Table 2) the estimated mean changes (95% CI) in BMI (kg/m^2^) over the 6-year period were 0.00 (referent), 0.26 (0.08, 0.44) and 0.39 (0.23, 0.56) for servings of <1/mo, 2–4/mo and **≥**1/week, respectively.

We also assessed the effects of including non-respondents at visit 3 who were alive at the time of visit 3, after imputing missing data. The results were similarly not much changed. For instance, for the full-model prospective analysis (equivalent to 3rd row in Table 2) the estimated 6-year increase in BMI (kg/m^2^) was 0.00 (referent), 0.24 (0.17, 0.47) and 0.38 (0.13, 0.63) for servings of <1/mo, 2–4/mo and **≥**1/week, respectively. There were 10,158, 7,277 and 13,294 participants who provided data in each of the three chocolate-intake categories.

## Discussion

Our main finding is that in the ARIC cohort more frequent consumption of chocolate was significantly associated with long-term greater weight gain. This association followed a dose-response-like pattern, with the greatest weight gain seen in participants with the highest frequency of chocolate intake. For instance, compared to participants who ate a chocolate serving less often than monthly, those who ate it 1–4 times a month and at least weekly experienced an increase in BMI (kg/m^2^) of 0.26 (95% CI: 0.08, 0.44) and 0.39 (0.23, 0.55), respectively, during the six-year study period. For participants of average height (1.68 m) these BMI increases are equivalent to a body weight gain (kg) of 0.73 (95% CI: 0.23, 1.24) and 1.10 (0.65, 1.58), respectively.

Our finding of a direct association between chocolate intake and weight gain is consonant with the results of a recent randomized-trial that a higher dose of chocolate led to a larger weight gain over a period of three months [16]. In addition to the high caloric density of chocolate, our results could also be partly due to decreased satiety induced by the regular intake of chocolate, as observed in a recent randomized controlled trial [17].

While our prospective analysis yielded a significant dose-response association between chocolate intake and increases in BMI over time, our cross-sectional analysis yielded the opposite: an inverse association between chocolate intake and current BMI. Our post-hoc subgroup analyses performed pursuant to finding significant interaction effect for prevalent obesity-related illness in our cross-sectional analysis (results are in Table 3) support our a priori hypothesized explanation for this difference between our prospective and cross-sectional findings: that subjects with high BMI who were diagnosed with obesity-related illnesses tended to reduce their intake of energy-rich foods, including chocolate, in an attempt to improve their prognosis - and thereby caused the observed inverse cross-sectional association between chocolate intake and BMI. We found that obese subjects who had developed an obesity-related illness between visit 1 and 3 exhibited a substantial decrease in chocolate intake (by ∼36%), sizeable increases in fruit (by ∼20%) and vegetables (by ∼17%) intake, and a slight decrease in BMI (by ∼2.6%, between visit 1 and 3 (Table 4). We also found significantly lower chocolate intake and higher BMI among participants with a prevalent obesity-related illness at visit 1. While these findings all support our hypothesis, the fact that they were uncovered in post hoc analyses requires that they be interpreted with caution. They require confirmation.

Golomb et al [3] found an inverse cross-sectional association in participants without evidence of CVD and diabetes, so their finding appears not to be due to reverse causation. However, the age range in Golomb et al.’s study was 20–85 years, compared to 45–64 years in the ARIC Study. It is therefore possible that a portion of Golomb et al’s elderly participants experienced preclinical symptoms and attempted to reduce intake of chocolate and other calorically-rich foods so as to lose weight to prevent the progression of their symptoms. This explanation may also be partly responsible for the inverse association in Strandberg et al.’s [4] elderly cohort. Both O’Neil et al,’s [2] cohort and the ARIC cohort were samples of American adults, and both found evidence of an inverse cross-sectional finding for all their participants.

One of the strengths of our study is the precise sequential body weight and height measurements in the ARIC cohort, which allowed for use of linear mixed model techniques that yielded detailed results with high precision. Similarly ARIC’s rich variety of possible confounder variables allowed for testing of three models with different levels of confounder adjustments. One limitation of our study is that the exposure variable, chocolate intake, was self reported. The use of food-frequency data is widely regarded as reliable for ranking participants according to their dietary intake even though these data do suffer from relatively high levels of intra-individual variation that includes measurement error [18]. Measurement error in the exposure variable tends to move regression coefficients closer to the null, so that it is likely that our significant estimates of the prospective BMI increases or cross-sectional BMI levels associated with different levels of chocolate intake would have been further from the null if we had a source of chocolate intake free of measurement error. Another limitation is that our exposure variable did not distinguish between different types of chocolate (white, milk, dark) so we were unable to assess the effects of chocolate type. There is some evidence that the flavanol epicatechin in cocoa can increase mitochondrial biogenesis in rodents [19], so if we had had data on chocolate type, we could have tested the hypothesis that dark chocolate yields smaller prospective BMI increases than milk or white chocolate in the ARIC cohort.

In conclusion, this epidemiological analysis in the prospective ARIC cohort found that a chocolate habit was associated with greater long-term weight gain in a dose-response pattern. Our cross-sectional finding that chocolate was associated with lower body weight only applied to participants with preexisting serious obesity-related illness.
